# Coronary Magnetic Resonance Angiography in Chronic Coronary Syndromes

**DOI:** 10.3389/fcvm.2021.682924

**Published:** 2021-08-17

**Authors:** Reza Hajhosseiny, Camila Munoz, Gastao Cruz, Ramzi Khamis, Won Yong Kim, Claudia Prieto, René M. Botnar

**Affiliations:** ^1^School of Biomedical Engineering and Imaging Sciences, King's College London, London, United Kingdom; ^2^National Heart and Lung Institute, Imperial College London, London, United Kingdom; ^3^Department of Cardiology and Institute of Clinical Medicine, Aarhus University Hospital, Skejby, Denmark; ^4^Escuela de Ingeniería, Pontificia Universidad Catolica de Chile, Santiago, Chile; ^5^Instituto de Ingeniería Biologica y Medica, Pontificia Universidad Catolica de Chile, Santiago, Chile

**Keywords:** coronary angiography, CMRA, CCS, atherosclerosis, plaque, magnetic resonance imaging

## Abstract

Cardiovascular disease is the leading cause of mortality worldwide, with atherosclerotic coronary artery disease (CAD) accounting for the majority of cases. X-ray coronary angiography and computed tomography coronary angiography (CCTA) are the imaging modalities of choice for the assessment of CAD. However, the use of ionising radiation and iodinated contrast agents remain drawbacks. There is therefore a clinical need for an alternative modality for the early identification and longitudinal monitoring of CAD without these associated drawbacks. Coronary magnetic resonance angiography (CMRA) could be a potential alternative for the detection and monitoring of coronary arterial stenosis, without exposing patients to ionising radiation or iodinated contrast agents. Further advantages include its versatility, excellent soft tissue characterisation and suitability for repeat imaging. Despite the early promise of CMRA, widespread clinical utilisation remains limited due to long and unpredictable scan times, onerous scan planning, lower spatial resolution, as well as motion related image quality degradation. The past decade has brought about a resurgence in CMRA technology, with significant leaps in image acceleration, respiratory and cardiac motion estimation and advanced motion corrected or motion-resolved image reconstruction. With the advent of artificial intelligence, great advances are also seen in deep learning-based motion estimation, undersampled and super-resolution reconstruction promising further improvements of CMRA. This has enabled high spatial resolution (1 mm isotropic), 3D whole heart CMRA in a clinically feasible and reliable acquisition time of under 10 min. Furthermore, latest super-resolution image reconstruction approaches which are currently under evaluation promise acquisitions as short as 1 min. In this review, we will explore the recent technological advances that are designed to bring CMRA closer to clinical reality.

## Introduction

Coronary artery disease (CAD) is the leading cause of cardiovascular morbidity and mortality worldwide (1, 2). CAD is predominantly caused by coronary atherosclerosis, which initiates in the early decades of life and progresses at a variable pace depending on the intrinsic propensity of the individual in combination with lifestyle modifications and therapeutic interventions (3–5).

The clinical manifestations of CAD can present either as an acute process or a more stable but chronic deterioration of symptoms. The European Society of Cardiology has recently categorised CAD as either acute coronary syndromes (ACS) or chronic coronary syndromes (CCS) (6). Whilst cardiovascular magnetic resonance (CMR) has a clear role in the diagnosis and management of patients with ACS, this is beyond the scope of this review. We will instead focus on the role of coronary magnetic resonance angiography (CMRA) in patients with CCS with a particular emphasis on the current status quo and future directions.

Chronic coronary syndromes can present clinically in a variety of ways; symptoms of stable angina (e.g., chest pain and/or dyspnoea), symptoms of heart failure or asymptomatic left ventricular impairment, symptomatic and asymptomatic cardiac arrhythmias (6). The early detection of CCS and targeted risk stratification will facilitate the timely initiation of therapeutic interventions and monitoring of disease progression.

X-ray coronary angiography is the reference standard imaging modality for the assessment of CAD with unrivalled temporal and spatial resolution, as well as the versatility to enable real time coronary intervention for patients presenting with ACS. The addition of functional physiological assessment of CAD with pressure wiring is particularly helpful in patients presenting with CCS and has been shown to be prognostically significant (7, 8). Plaque characterisation and coronary vascular anatomy can be further complemented with intravascular imaging (9, 10). However, risk from ionising radiation, viscous iodine-containing radiocontrast agent induced acute kidney injury and its potential progression to chronic kidney disease (11) as well as potential invasive complications limit the surveillance suitability of invasive assessment for CCS beyond the initial diagnosis.

Computed tomography coronary angiography (CCTA) has emerged as a credible non-invasive alternative for the assessment of patients with CCS. It offers high diagnostic accuracy in terms of comprehensive assessment of luminal stenosis, fractional flow reserve and plaque characterisation (12–15). However, it is also limited by ionising radiation, iodinated nephrotoxic contrast agents and calcium related blooming artefacts, dampening its suitability in patients with highly calcified plaque lesions or for the longitudinal assessment of CAD.

Coronary magnetic resonance angiography could potentially offer a non-invasive alternative for the early detection and long-term monitoring of CCS, which is free of ionising radiation and iodinated nephrotoxic contrast agents. It could also be combined with volumetric assessment of left ventricular function, myocardial perfusion, myocardial tissue characterisation, valvular assessment and coronary plaque characterisation to brand CMR as one of the most comprehensive imaging modalities for cardiovascular assessment. Here we discuss the current status quo and potential future role of CMRA as a viable alternative for the assessment of patients with CCS.

## Coronary Magnetic Resonance Angiography

The potential of CMRA to exclude significant CAD was first demonstrated two decades ago in a multicentre study of 109 patients, which compared non-contrast CMRA against invasive X-ray angiography (16). In this study, the sensitivity, specificity, positive and negative predictive values, and accuracy of CMRA were 93, 42, 70, 81, and 72% respectively. These findings were confirmed in a subsequent study of 127 patients comparing CMRA vs. invasive X-ray angiography (17). In this study, the sensitivity, specificity, positive and negative predictive values, and accuracy of CMRA were 88, 72, 71, 88, and 79% respectively. In a direct head-to-head comparison of CMRA vs. CCTA in patients with suspected CAD who were also assessed with invasive X-ray coronary angiography, there was no significant difference between CCTA and CMRA in terms of diagnostic accuracy, suggesting that CMRA could be used in these patients to exclude significant disease, inform revascularization and if the CMRA scan was negative the event free survival rate was comparable with CCTA (18, 19). To assess the prognostic value of CMRA, 207 patients with suspected CAD who underwent non-contrast whole-heart CMRA were followed up by Yoon et al. (20). Coronary stenosis was significantly associated with major adverse cardiac events (myocardial infarction, cardiac death, and unstable angina) and all cardiac events (which included revascularization >90 days after CMRA).

However, despite the early promise of CMRA, clinical application is currently confined to a few specialised centres within a set of niche indications e.g., suspected anomalous coronary arteries, coronary artery aneurysms and assessment of the proximal coronary arteries. Reasons for the slow uptake of CMRA in clinical practise include lower spatial resolution compared to CCTA and invasive X-ray angiography, significantly longer and unpredictable acquisition time, complicated scan planning, and motion related (cardiac and respiratory) image quality degradation.

Significant technological strides in CMR image acquisition and image processing are now enabling some of these technical challenges to be overcome. The most notable breakthroughs are in the fields of respiratory motion compensation and image acceleration. Further recent advances in deep learning-based motion correction and image reconstruction could potentially enable 3D whole-heart CMRA with similar spatial resolution and acquisition time as CCTA in the future. We will discuss some of these recent developments in the following sections.

## Cardiac and Respiratory Motion Compensation

In order to acquire a high spatial resolution 3D whole-heart CMRA, patients are required to be continuously scanned for several minutes with data acquired from numerous heat beats under free breathing. This scenario produces a challenging multi-dimensional cardiac and respiratory motion environment, which needs to be compensated through image acquisition and reconstruction techniques. Cardiac motion artefacts are typically minimised by prospective electrocardiographic (ECG) triggering to acquire data during mid-to-late diastole. However, this may be susceptible to image degradation as a result of cardiac arrhythmias, heart rate variability or user dependent estimation of the resting period. Systolic image acquisition is less sensitive to arrhythmias and heart rate variability. However, longer acquisition times may be needed due to a shorter resting period compared to diastolic imaging. Alternatively, data can be continuously acquired and then retrospectively reconstructed into multiple cardiac phases, thus selecting the phase with the minimum cardiac motion artefact, similar to CCTA (21, 22).

Various techniques have been proposed to compensate for respiratory motion artefacts during CMRA acquisition. The most rudimentary techniques utilise breath-holding to minimise respiratory motion artefacts (23–26), which enable both 2D and 3D CMRA acquisitions within a single albeit long breath-hold (27–29). However, image quality is frequently suboptimal due to diaphragmatic drift and difficulty of patients to hold their breath for a prolonged period (30, 31). As a result, clinically established 3D CMRA techniques implement free-breathing motion compensated protocols (30, 32). Initially, respiratory bellows (33) and amplitude demodulation of the ECG signal (34), which are external respiratory monitoring devices were used to enable free breathing CMRA. This is called respiratory gating as data is only acquired at end expiration when respiratory motion is minimal. However, this method is both labour intensive and time consuming to implement. Furthermore, it is not possible to estimate the true multidimensional extend of respiratory motion. As a result, respiratory navigators, incorporating the intrinsic CMR signal to track respiratory related movement of the heart became the dominant form of respiratory gating/compensation for free breathing CMRA. Here, the diaphragmatic 1D navigator (35, 36) takes advantage of the liver-diaphragm interface to perform respiratory translational motion estimation, which enables both respiratory gating and 1D (superior-inferior) motion correction. However, there is a non-linear relationship (37) between the movement of the liver/diaphragm and the heart, which is patient specific and subject to hysteresis (the motion of breathing-in is different than breathing-out). Despite this, a population derived linear correction factor of 0.6 is used as an approximation of the motion between liver/diaphragm and the heart (38, 39). Furthermore, precise planning of the diaphragmatic navigator is complex and requires expertise, acquisition times are often unpredictable and reliant on the breathing pattern of patients and it is highly inefficient (~30–50%) as data is acquired only at end expiration within a very narrow acquisition window of ±5 mm (30, 36).

**Figure 1:**
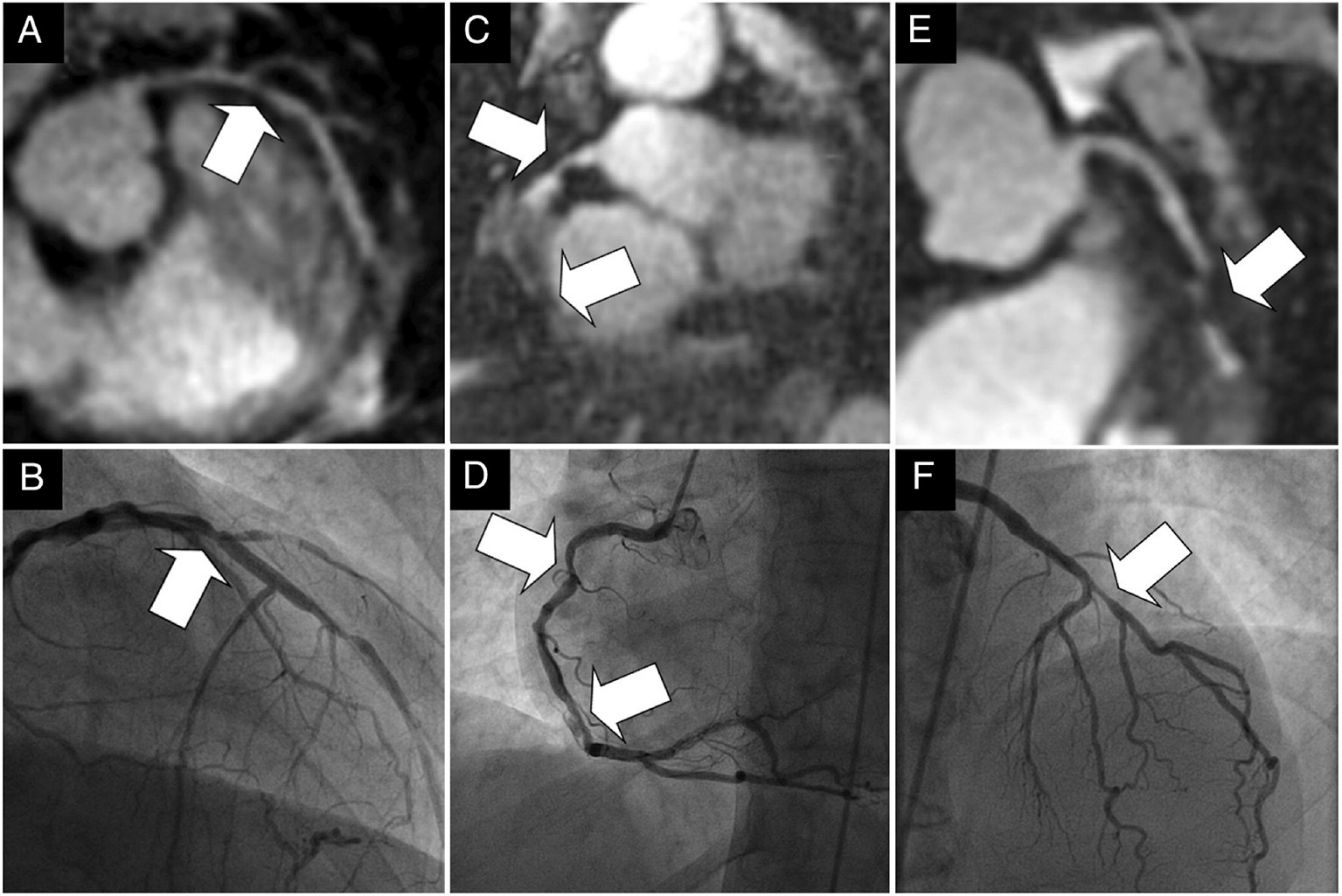
**(A–F)** Examples of the comparison between multiplanar reformats of the whole-heart 1D self-navigated CMRA data sets (top row) and the corresponding x-ray coronary angiograms (bottom row) in three patients. The lesion in the proximal LAD artery and just distal to the take-off of a diagonal branch can clearly be identified in the first patient in **(A)**. while this is confirmed on the x-ray angiogram in **(B)**. While the luminal narrowing of the proximal RCA in the second patient on **(C)**. can clearly be identified, the further course of this artery is obscured in the region of a stent. The same in stent restenoses can be identified in **(D)**. In the third patient in **(E)**. significant disease is identified in the proximal LAD artery at CMRA and is confirmed on, **(F)**. the corresponding x-ray coronary angiogram. Arrows = stenoses; LAD, left anterior descending artery; RCA, right coronary artery. Adapted with permission from Piccini et al. (46).

Several novel respiratory motion compensation frameworks have recently been proposed to overcome some of these drawbacks, principally to deal with the 3D nature of respiratory motion, improve image quality, increase respiratory scan efficiency to 100% and therefore significantly reduce acquisition times. Using the so called “1D self-navigation” approach (40), the acquired CMR data is used to estimate displacement/movement of the heart induced by respiratory motion (40, 41), eliminating the need for the correction factor, enabling translational motion correction and acquiring data at every point of the respiratory cycle which enables 100% respiratory scan (42–45). In a cohort of 78 patients, a self-navigated CMRA framework enabled 92, 84, and 56% of proximal, middle and distal coronary segments, respectively, to be visualised, with a per vessel sensitivity and specificity for stenosis (>50%) detection of 65 and 85% compared with X-ray coronary angiography (Figure 1) (46).

**Figure 2:**
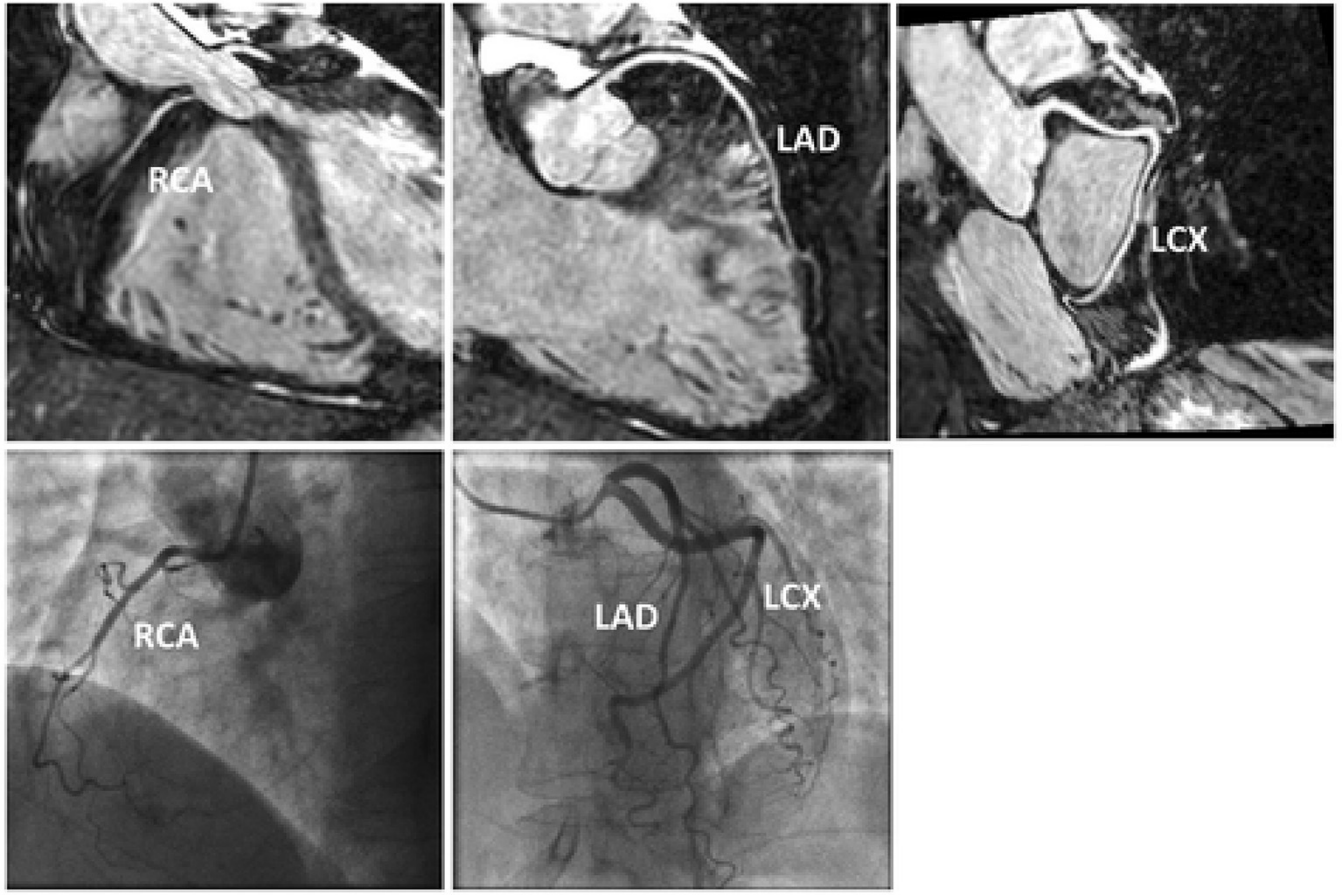
Reformatted CMRA datasets (top row) from a patient without coronary artery disease but non dominant RCA. Coronary x-ray angiography in the same patient (bottom row). RCA, right coronary artery; LAD, left anterior descending artery; LCX, left circumflex artery. Adapted with permission from Henningsson et al. (52).

However, with 1D self-navigation, it is difficult to separate moving (e.g., heart) from static (e.g., chest wall) tissue, which introduces artefacts (47). Image-based navigators (iNAVs) are an alternative platform to respiratory self-navigation. Whilst offering up to 100% respiratory scan efficiency, the principle aim of iNAVs is to separate moving tissue from static tissue by acquiring a low spatial resolution 2D/3D image at every heartbeat prior to the acquisition of high resolution CMRA (47–51). This framework also enables the capability to estimate respiratory motion in multiple directions to factor in the multidimensional motion of the heart. Furthermore, it eliminates additional planning as the iNAV can be derived within the same field of view (FOV) and orientation as the CMRA planning. An early version of iNAV CMRA framework demonstrated highly diagnostic image quality in the proximal, middle and distal coronary segments (98, 94, and 91, respectively) (Figure 2) (52). The sensitivity, specificity, and negative predictive values were 86, 83, and 95% per patient, 80, 92, and 97% per vessel and 73, 95, and 98% per segment; compared with X-ray coronary angiography.

## Coronary Magnetic Resonance Angiography Techniques Currently in Development

Despite offering 100% respiratory scan efficiency, self-navigation or iNAV frameworks on their own are not sufficient. It can take up to 30 min to acquire a fully sampled high resolution (≈1 mm isotropic) CMRA. Here we will discuss the novel techniques in development which incorporate self-navigation or iNAV systems with a combination of image acceleration techniques (undersampled acquisition, parallel imaging, iterative non-linear reconstruction) to achieve high-spatial resolution CMRA within a clinically feasible acquisition time. We will also touch on more advanced motion correction frameworks which factor in the more complex 3D non-rigid motion of the heart within the thoracic cavity. Finally, we will briefly discuss emerging deep learning super resolution CMRA reconstruction frameworks that could potentially enable 3D whole-heart CMRA with similar spatial resolution and acquisition time as CCTA.

**Figure 3:**
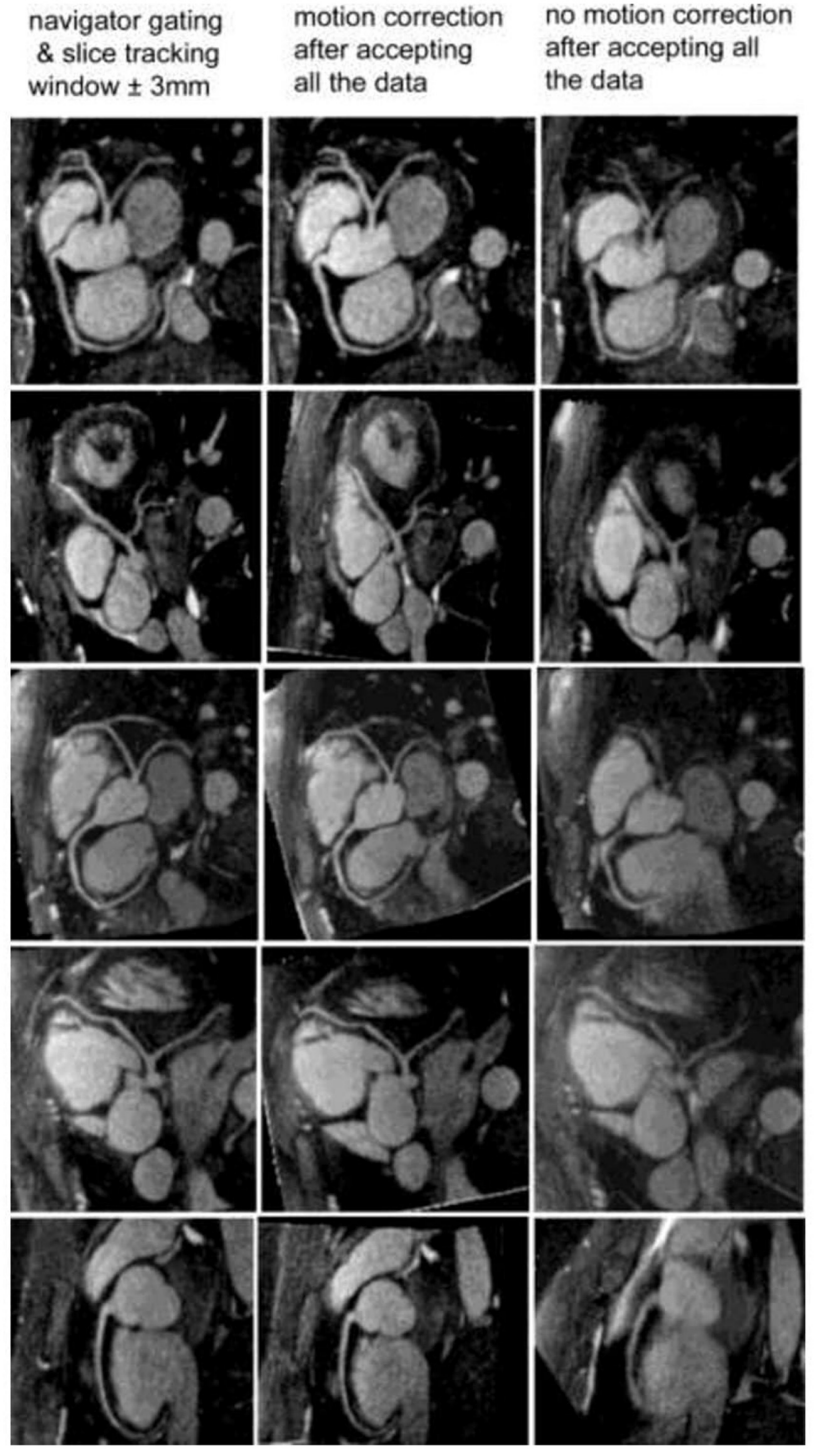
Reformatted coronary artery images from five healthy volunteers. With the motion correction technique (middle column), coronary artery visualization is excellent and similar to the navigator gating and slice tracking approach (left column). Without any motion correction (right column), the images are blurry and the coronary artery visualization is poor. The imaging time with the motion correction technique is reduced by a factor of 2.5 to 3 compared with the navigator gating and slice tracking approach. Adapted with permission from Bhat et al. (53).

**Figure 4:**
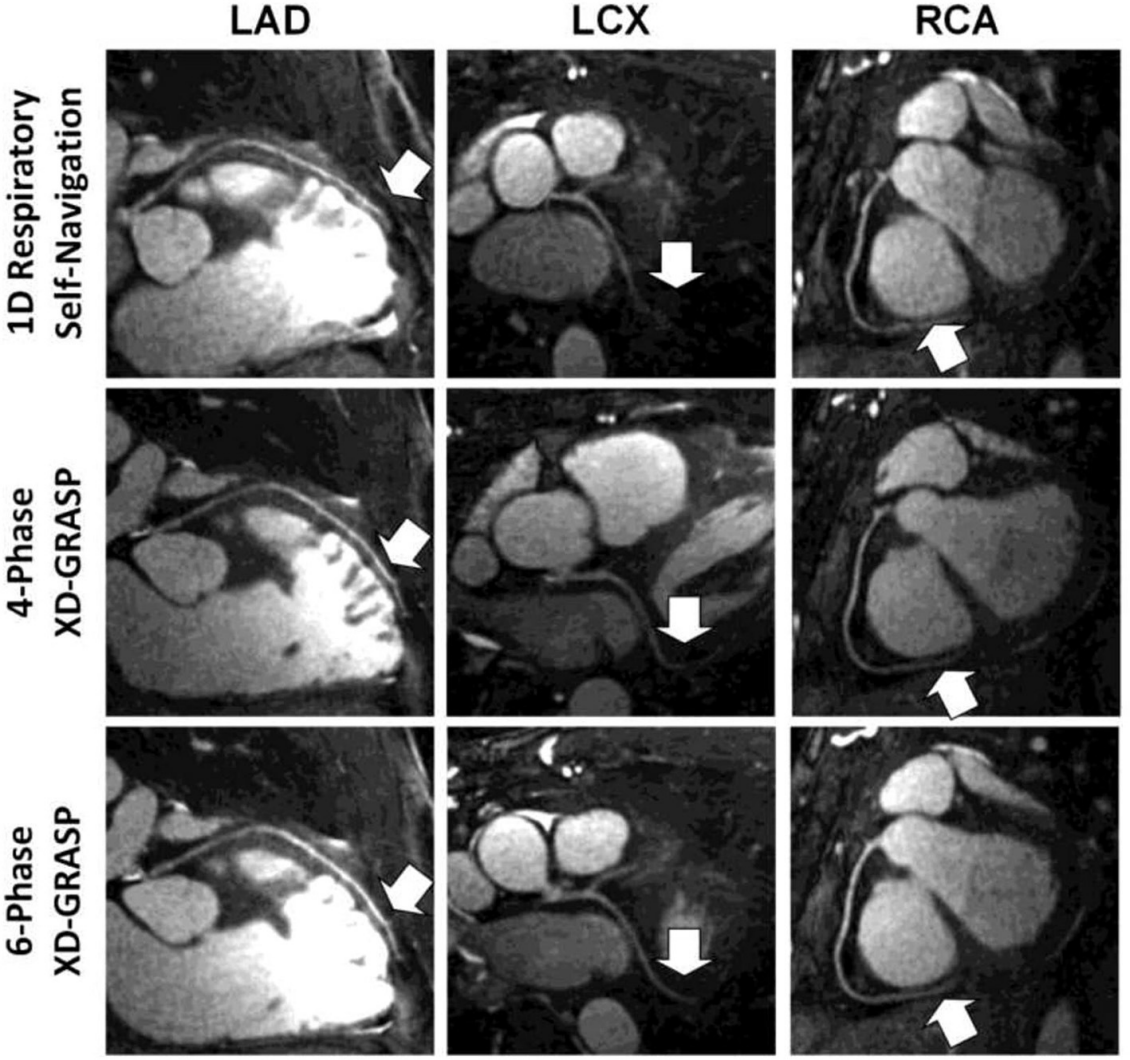
Examples of multiplanar reformatted coronary arteries from one representative healthy adult volunteer. Although respiratory self-navigated reconstruction with 1D motion correction could achieve high image quality (top row), a clear improvement in sharpness as well as visible vessel length (arrows) can be noticed in both four-phase (middle row) and six-phase (bottom row) extradimensional golden-angle radial sparse parallel (XD-GRASP) reconstructions. Adapted with permission from Piccini et al. (54).

**Figure 5:**
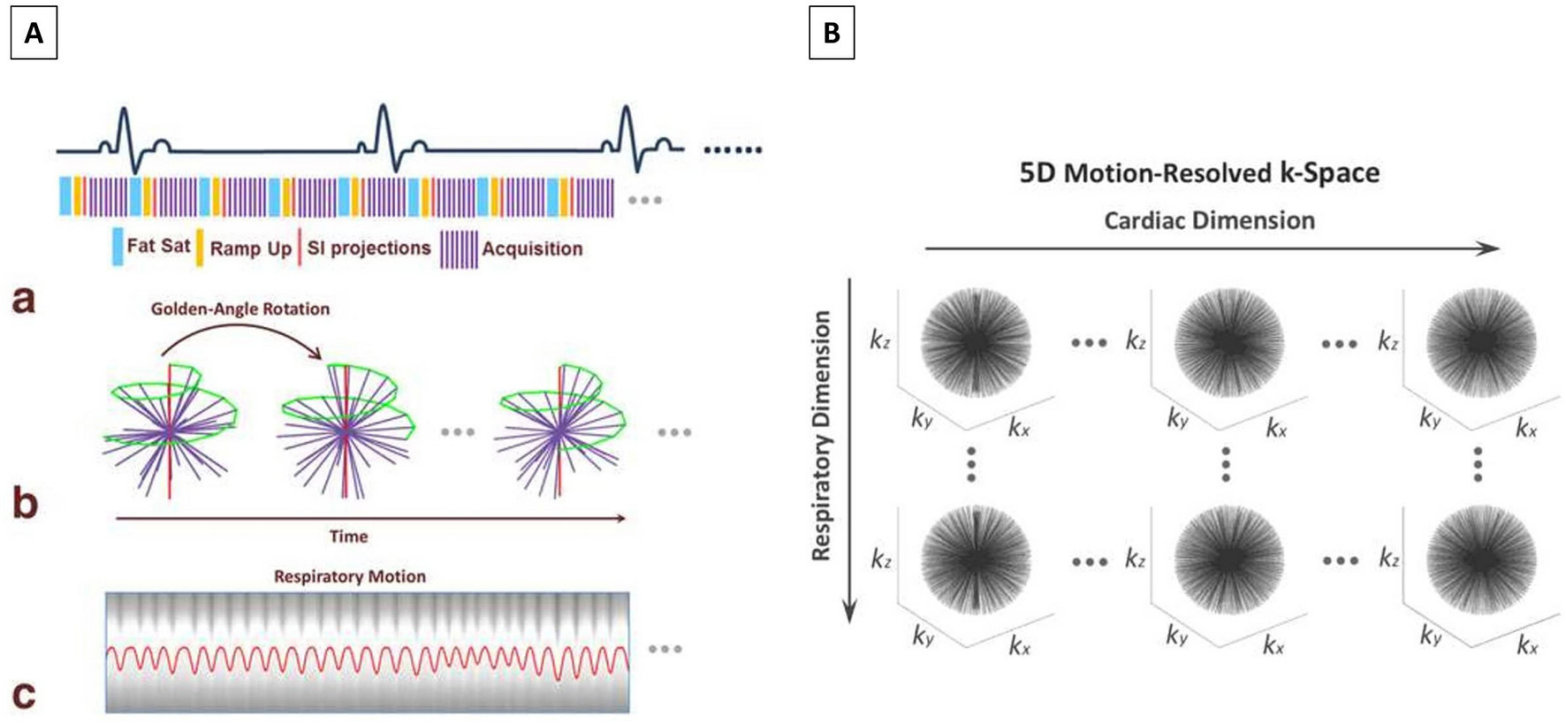
**(A)** Data acquisition scheme and respiratory motion extraction for non–ECG-triggered whole-heart imaging. (a) A 3D radial b-SSFP sequence that is segmented into multiple interleaves (purple lines) is used for MR data acquisition. Each interleave starts with a spoke oriented along the superior to inferior direction for self-navigation (red lines) and is preceded by fat saturation (blue lines). Ten additional ramp-up RF pulses (yellow lines) are deployed between the fat saturation module and the data acquisition window to restore restoring steady-state at each interleave. (b) 3D radial sampling trajectory based on the spiral phyllotaxis pattern. Each interleave is rotated by the golden-angle (137.51 °) about the z-axis, starting with a self-navigation spoke (red lines) for respiratory motion extraction. (c) An extracted respiratory motion signal is superimposed to the 1D FFT of an example series of SI readouts. **(B)** The acquired k-space is sorted into a 5D dataset (*k*_*x*_*-k*_*y*_*-k*_*z*_*-*cardiac phase-respiratory phase) using respiratory motion signal extracted from self-navigators and cardiac motion signal obtained from recorded ECG time stamp. The datasets are first binned into different cardiac phases with a desired temporal resolution, then each cardiac phase is further sorted into multiple respiratory motion phases spanning from end-expiration to end-inspiration. The data sorting process is performed such that the number of spokes grouped in each temporal phase is the same. SSFP, Steady State Free Precession; MR, Magnetic Resonance; RF, Radiofrequency; FFT, fast Fourier transform; SI, Superior-Inferior; ECG, Electrocardiogram. Adapted with permission from Feng et al. (55).

**Figure 6:**
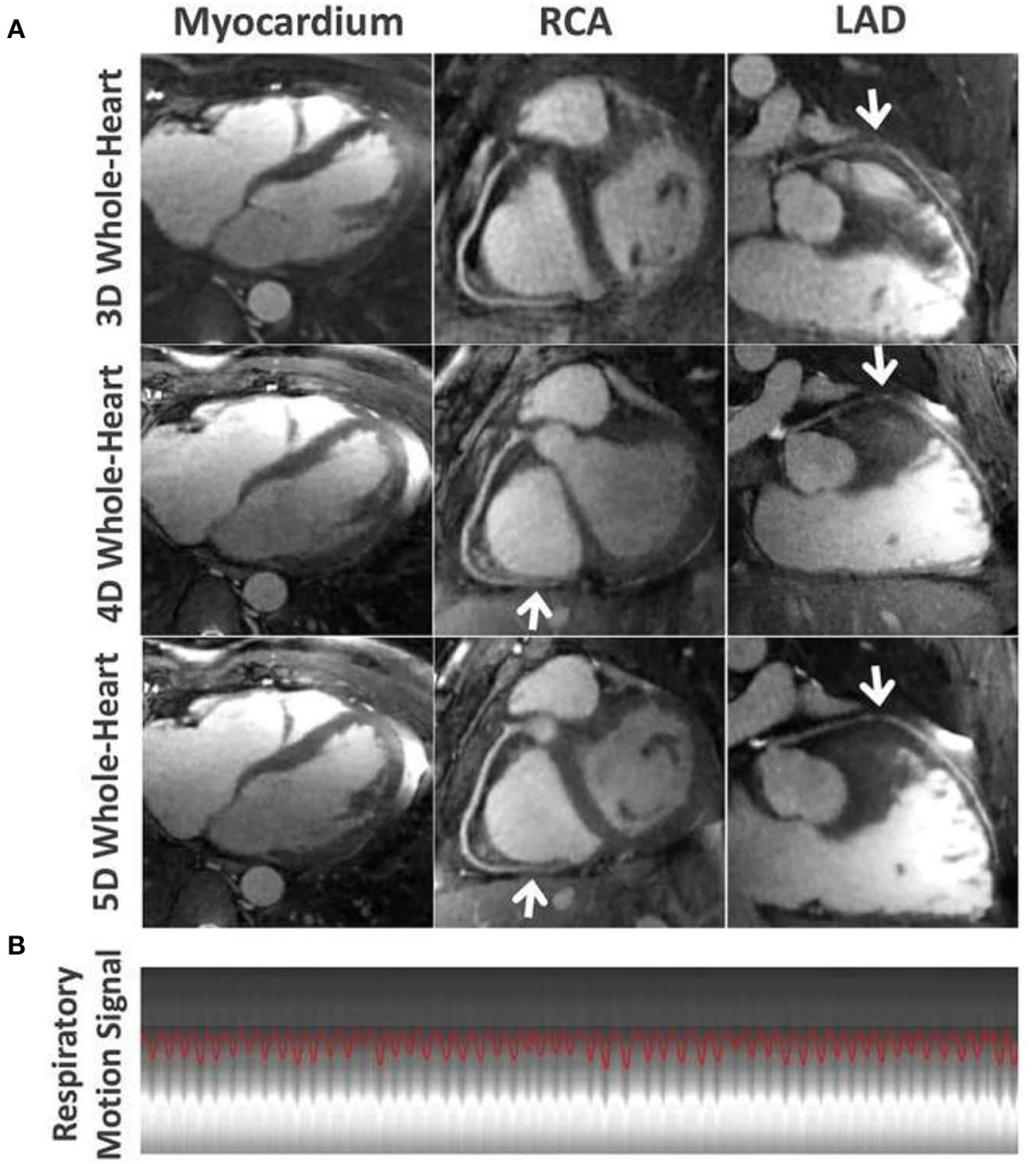
**(A)** Comparison of the myocardium, the RCA and LAD coronary arteries for different imaging techniques in one subject. 5D whole-heart images (end-expiratory phase) achieved improved visual delineation of the myocardial wall and different segments of the coronary arteries (white arrows) over 4D whole-heart images, and improved delineation of the LAD over self-navigated 3D whole-heart images. **(B)** Corresponding respiratory motion pattern extracted from the continuous acquired whole-heart dataset in this subject. RCA, right coronary artery; LAD, left anterior descending artery. Adapted with permission from Feng et al. (55).

It is possible to combine an undersampled 3D radial trajectory acquisition with 1D navigators (diaphragmatic or self-navigation) to allocate the acquired data into specific phases in the respiratory cycle (respiratory bins). Registration algorithms subsequently assess the motion between each bin and a reference end-expiratory bin. Images from all respiratory bins are then motion corrected to the common respiratory position and averaged to produce a respiratory motion corrected image. This methodology allows for 100% respiratory scan efficiency with a drastically shorter acquisition time in comparison to respiratory gating alone with similar image quality (Figure 3) (22, 40, 41, 53). Respiratory resolved frameworks have been proposed to further reduce the multidirectional non-rigid motion related artefacts (Figure 4) (54), displaying substantial enhancement in vessel length depiction as well as vessel sharpness in comparison to 1D self-navigation frameworks. This approach has been extended to cardiac phases (so called “5D whole-heart”) from free-running frameworks (Figures 5, 6) (21, 55–57). However, lower signal to noise ratio and prolonged reconstruction time (with compressed sensing) are associated with radial sampling. Spiral trajectory k-space CMRA acquisitions have been proposed to improve scan efficiency, the signal to noise ratio and reduce undersampling artefacts by oversampling near the k-space origin (58–60).

**Figure 7:**
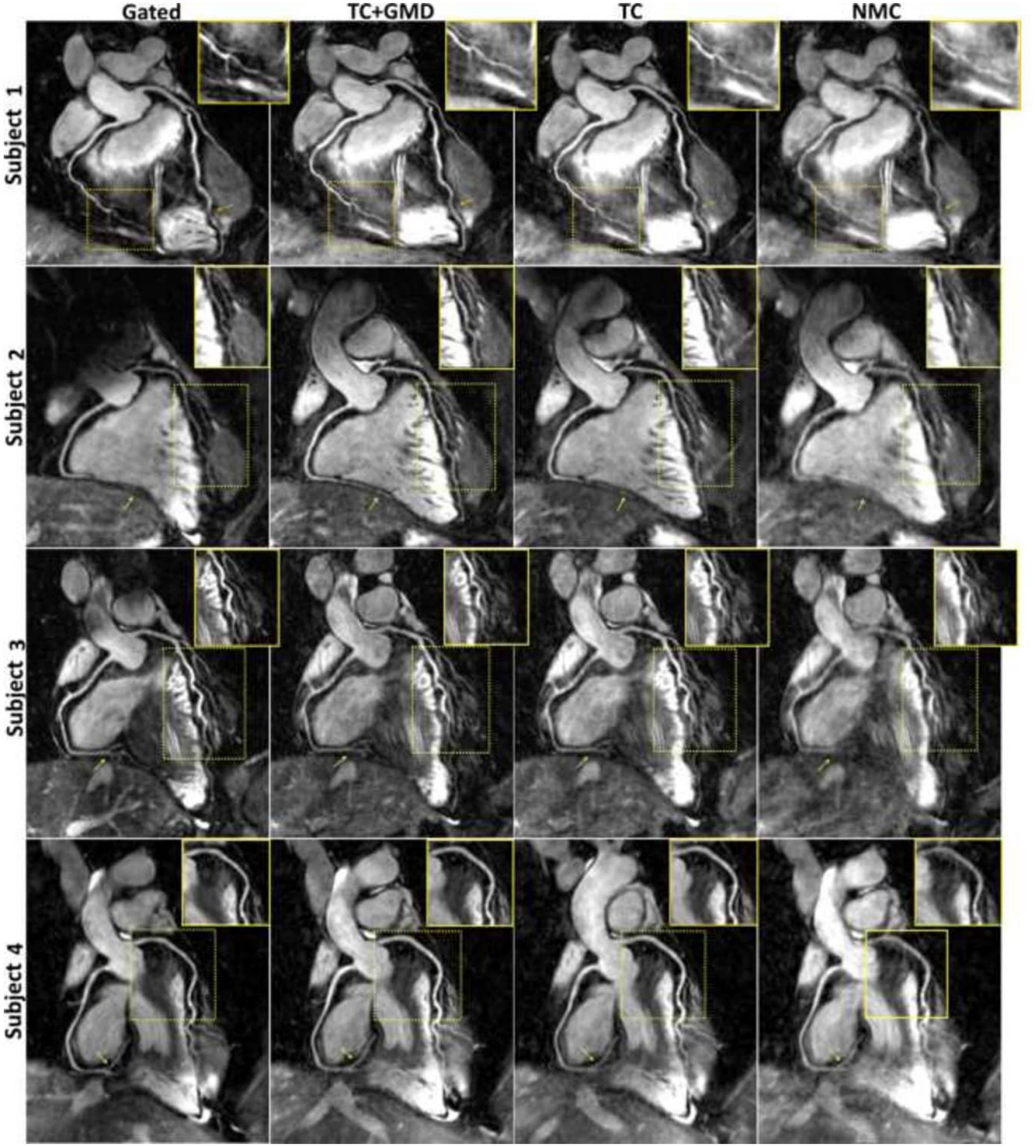
Reformatted coronary lumen images for gated, TC+GMD, TC, and NMC for subjects 1–4. Blurring present in the NMC images is reduced with TC, and sharpness is further increased with TC+GMD (magnified boxes). The distal part of both coronaries is particularly affected by motion (arrows). Note that TC and TC+GMD have image quality similar to that for gated. GMD, general matrix description; TC, translational correction; TC+GMD, two-step translational and non-rigid correction; NMC, non–motion-corrected. Adapted with permission from Cruz et al. (62).

**Figure 8:**
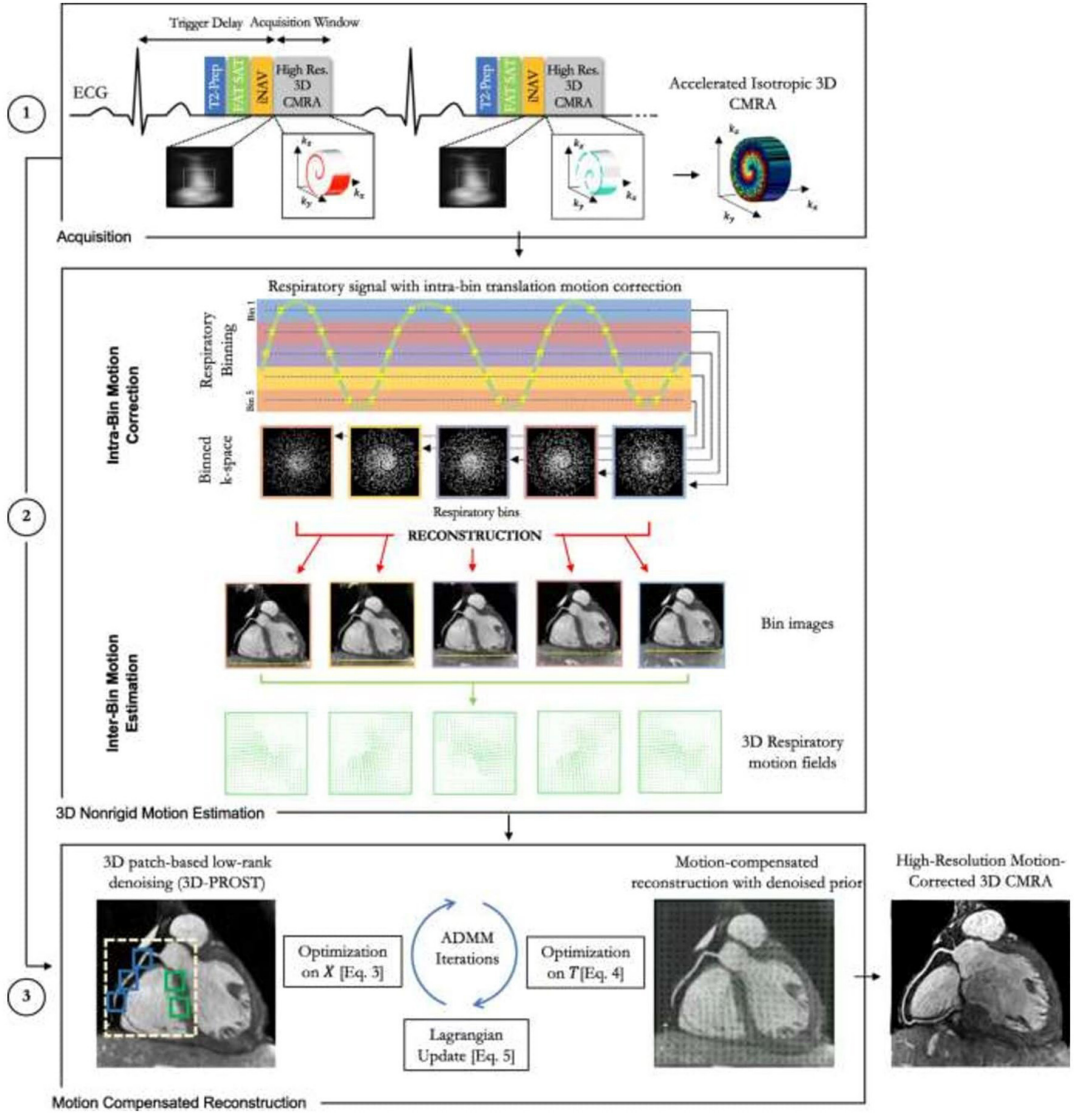
Schematic overview of the accelerated free-breathing 3D CMRA acquisition with sub-millimeter isotropic resolution, 100% scan efficiency and non-rigid motion-compensated PROST reconstruction. (**1**) CMRA acquisition is performed with an undersampled 3D variable density spiral-like Cartesian trajectory with golden angle between spiral-like interleaves (VD-CASPR), preceded by 2D image navigators (iNAV) to allow for 100% scan efficiency and beat-to-beat translational respiratory-induced motion correction of the heart. (**2**) Foot-head respiratory signal is estimated from the 2D iNAVs and used to assign the acquired data into five respiratory bins and translation-corrected respiratory bins. Subsequent reconstruction of each bin is performed using soft-gated SENSE and 3D non-rigid motion fields are then estimated from the five reconstructed datasets. (**3**) The final 3D whole-heart motion-corrected CMRA image is obtained using the proposed 3D PROST non-rigid motion-compensated reconstruction. CMRA, coronary magnetic resonance angiography; PROST, patch-based undersampled reconstruction; ADMM, alternating direction method of multipliers. Adapted with permission from Bustin et al. (65).

**Figure 9:**
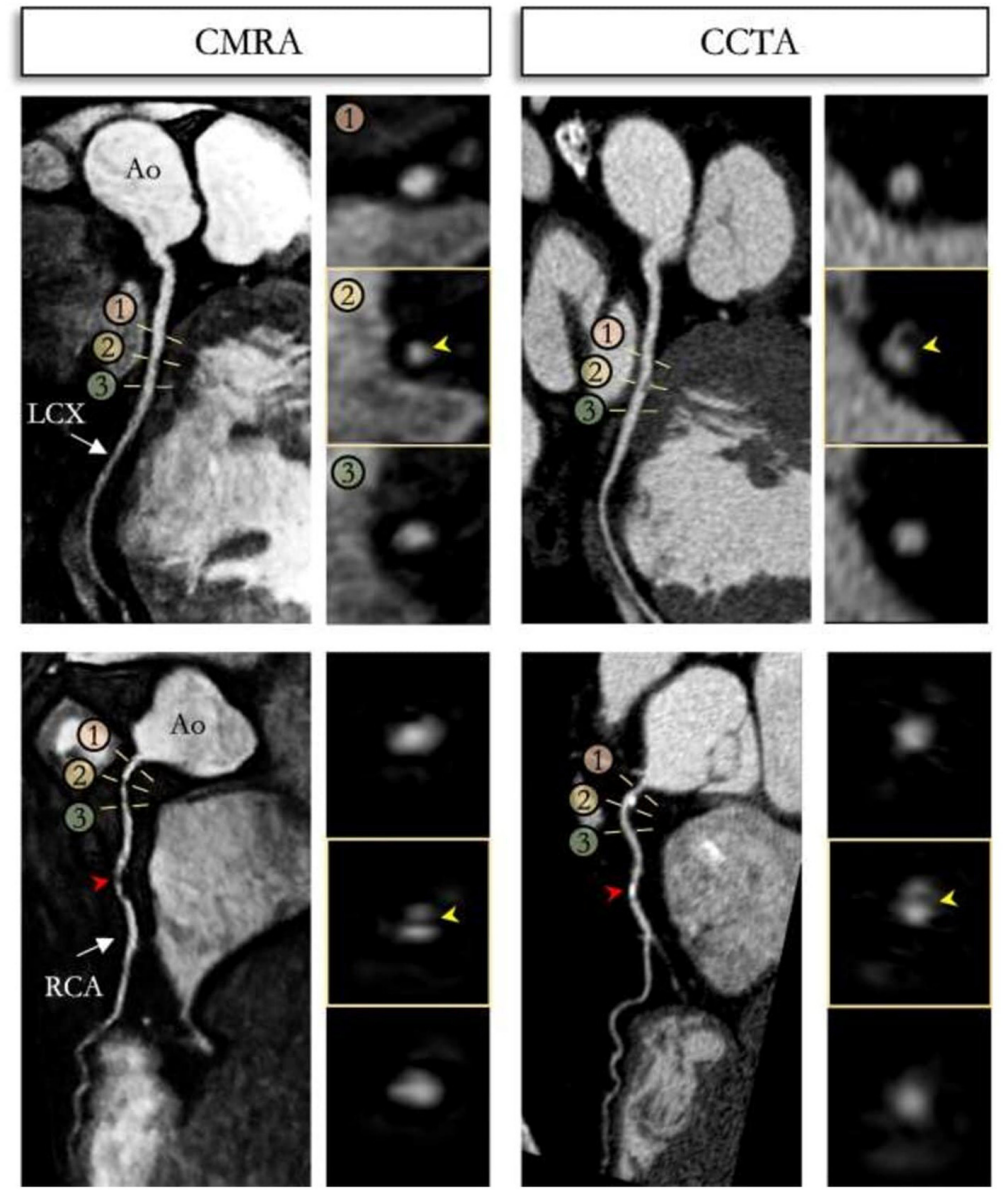
Reformatted non-contrast whole-heart sub-millimeter isotropic CMRA (left) and CCTA (right) images along the LCX (top) and RCA (bottom) are shown for a 54 year-old male patient. The CMRA dataset was acquired in 9 min with 100% scan efficiency (heart rate of 57 bpm). The CCTA images demonstrate mild (25–49%) disease with a calcified plaque within the proximal RCA and severe disease (70–90%) with a partially calcified plaque in the mid-segment of RCA (red arrows), and minimal (0–24%) disease with calcified plaque in the mid-segment of the LCX. Luminal narrowing is seen on the cross-sectional views at the sites of coronary plaque on the CMRA images (yellow arrows). LAD, left anterior descending artery; RCA, right coronary artery; LCX, left circumflex artery; Ao, aorta. Adapted with permission from Bustin et al. (65).

**Figure 10:**
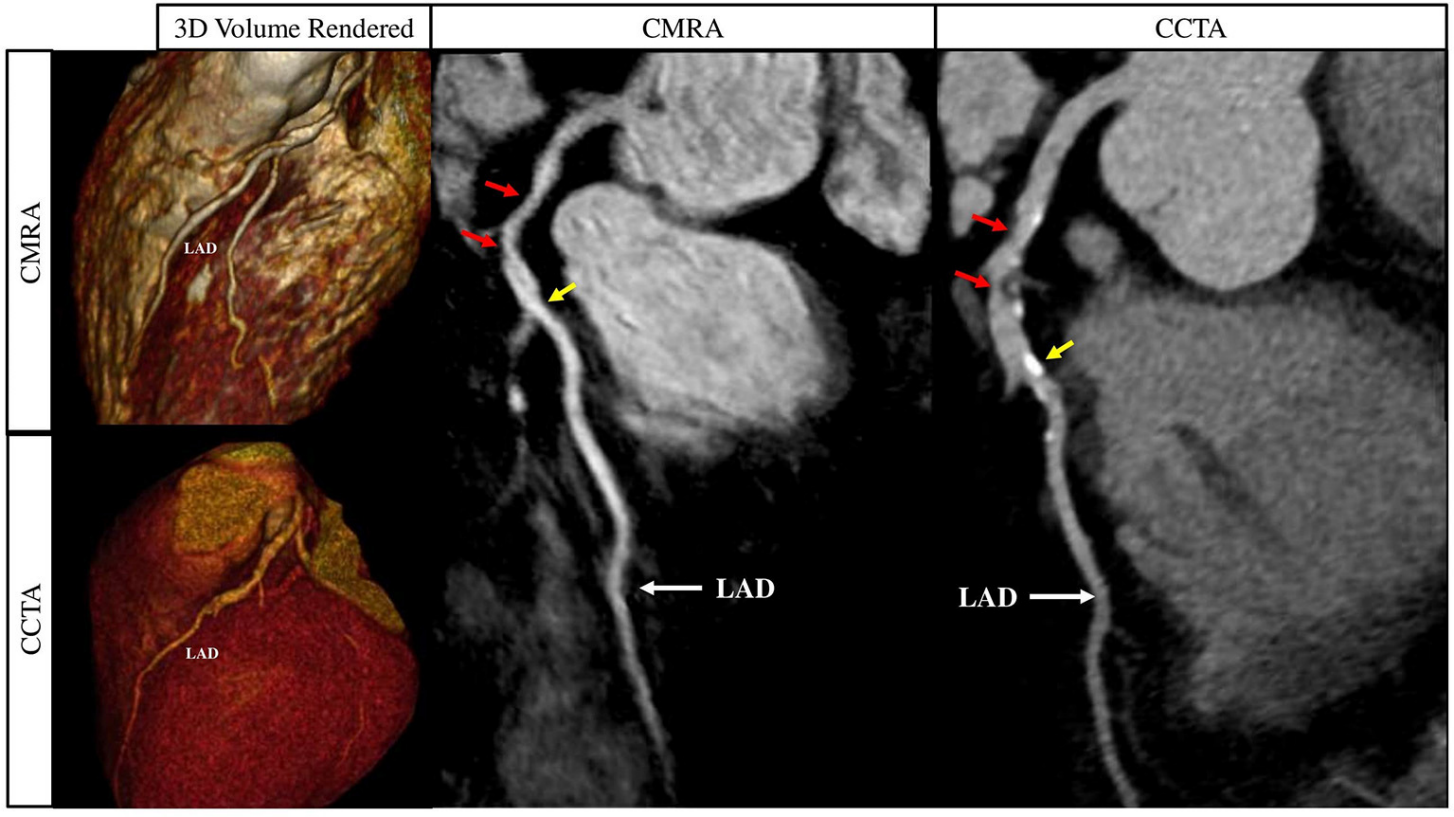
Curved multiplanar reformat and 3D volume rendered non-contrast CMRA and contrast enhanced CCTA in a 60 year old male with >50% partially calcified stenosis in the proximal to mid LAD on either side of the first diagonal artery (red arrows). The yellow arrows represent a focal calcified <50% stenosis just distal to the second diagonal artery. CMRA, Coronary Magnetic Resonance Angiography; CCTA, Coronary Computed Tomography Angiography; LAD, Left Anterior Descending Artery. Adapted with permission from Hajhosseiny et al. (66).

An alternative approach is a golden-step Cartesian trajectory with spiral profile ordering k-space acquisition, which can be combined with iNAVs to enable respiratory binning as described above (61). Using the binned data, it is possible to estimate and subsequently reconstruct the 3D non-rigid respiratory motion by combining beat-to-beat 2D translational and bin-to-bin 3D non-rigid motion correction to a common reference position (Figure 7) (62). This approach can increase the signal to noise ratio compared with radial sampling, whilst also improving image quality compared with 2D rigid translational motion correction frameworks. To shorten acquisition times and therefore enable higher spatial resolutions, Bustin et al. (63) adapted a highly undersampled patch-based CMR reconstruction framework (64) to propose a 3D patch-based low-rank (PROST) reconstruction, enabling <1 mm^3^ spatial-resolution free-breathing whole-heart 3D CMRA with <10 min predictable acquisition time with 100% scan efficiency. In a validation cohort of healthy subjects, image quality was comparable to the fully sampled acquisition and significantly improved compared to both iterative SENSE and compressed sensing reconstruction methods. This framework has been extended to include bin-to-bin non-rigid respiratory motion correction and has been compared against CCTA in patients with suspected CAD (Figures 8, 9) (65). In a single centre study of 50 patients, this CMRA technique obtained diagnostic image quality in 95, 97, 97, and 90% of all, proximal, middle and distal coronary segments, respectively. Furthermore, 100, 97, 96, and 87% of left main stem, right coronary artery, left anterior descending and left circumflex artery segments, respectively on CMRA were of diagnostic image quality (Figure 10) (66). The sensitivity, specificity, positive predictive value, negative predictive value and diagnostic accuracy were as follows: per patient (100, 74, 55, 100, and 80%), per vessel (81, 88, 46, 97, and 88%) and per segment (76, 95, 44, 99, and 94%), respectively, with an average acquisition time of 10.7 min at 0.9 mm isotropic spatial resolution (66).

**Figure 11:**
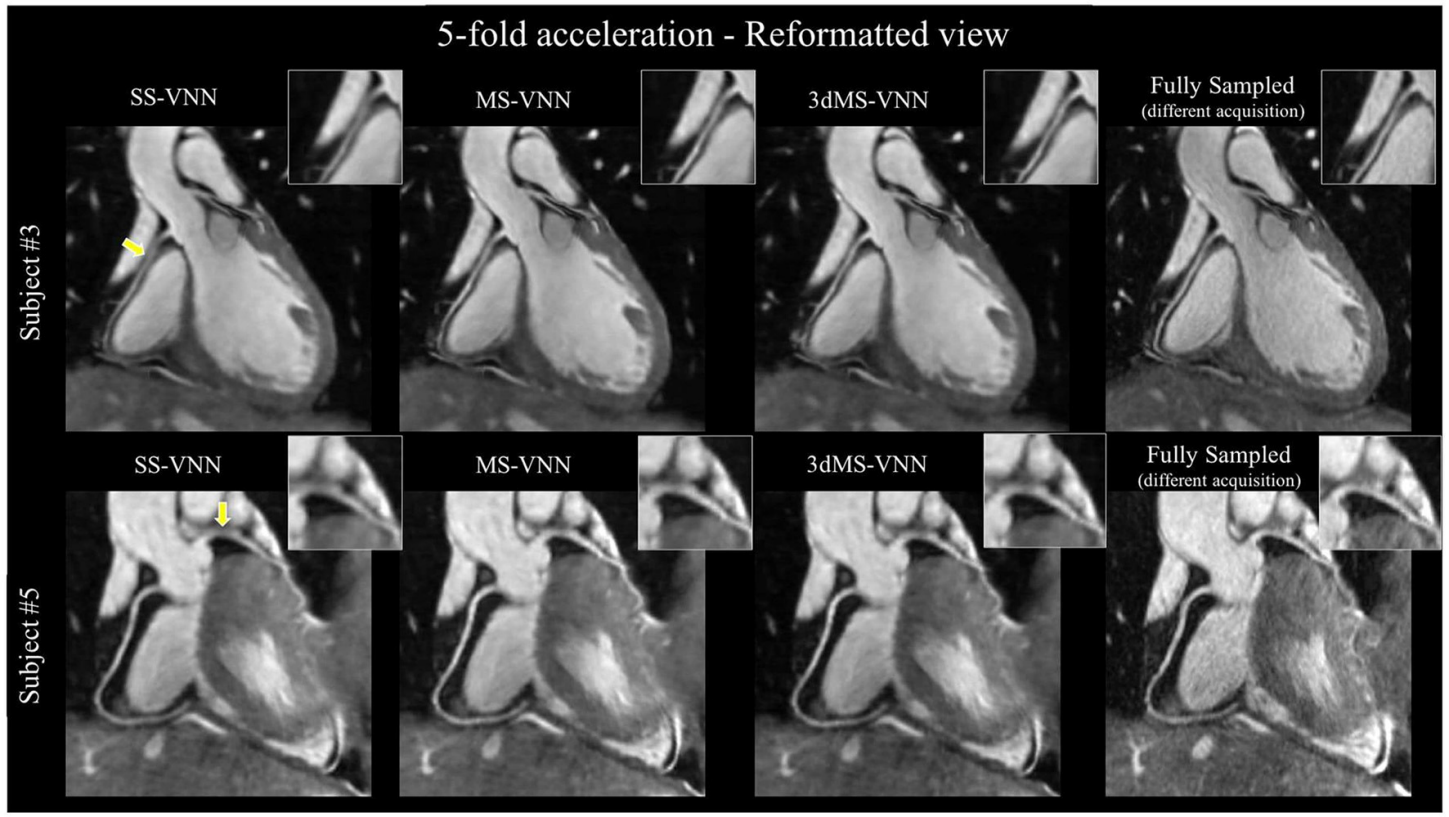
CMRA images reconstructed with a single-scale VNN (SS-VNN), the multi-scale VNN (MS-VNN), and a pseudo 3D multi-scale VNN (3dMS-VNN). CMRA images were reformatted along the left (LAD) and right (RCA) coronary arteries, for two representative healthy subjects. Fully sampled and undersampled acquisitions with acceleration factor of 5× are shown. VNN, variational neural network. Adapted with permission from Fuin et al. (72).

Whilst an acquisition time of ~10 min for sub-1 mm isotropic spatial resolution is clinically feasible, it is nevertheless considerably longer than a full CCTA scan (even when coronary artery calcium scoring and contrast enhanced coronary angiography are combined). It is particularly disadvantageous if image quality is sub-optimal on first attempt and repeat imaging within the same scan session is required. Furthermore, reconstruction times of iterative methods are relatively long and computationally demanding, especially if combined with non-rigid motion correction or non-Cartesian trajectories. Finally, highly undersampled acquisitions are vulnerable to residual aliasing or staircasing and blurring artefacts, putting a realistic limit on the extend of conventional image acceleration methods. Recently, deep-learning based image reconstruction networks have been proposed to address some of these shortcomings (67–71). The deep neural network scheme is trained to recognise prior reconstructed data samples retrospectively, which is then adapted to prospectively reconstruct acquired images in a matter of seconds (Figure 11) (72). However, whilst this scheme partially resolves the time-consuming image reconstruction process, it still relies on a prospectively acquired high spatial resolution CMRA acquisition. Recently, deep learning based super resolution schemes have been proposed to overcome this drawback. Here, a low spatial resolution image is prospectively acquired, often with low to intermediate acceleration factors, thereby significantly shortening the time the patient is on the scanning table, whilst simultaneously employing trained deep neural networks to reconstruct to a higher target spatial resolution within a few seconds (73–82). These novel deep learning schemes have the potential to finally unlock the true potential of CMRA in the clinical setting, which require further prospective clinical validation against CCTA and invasive X-ray angiography.

## Magnetic Resonance Coronary Plaque Imaging

**Figure 12:**
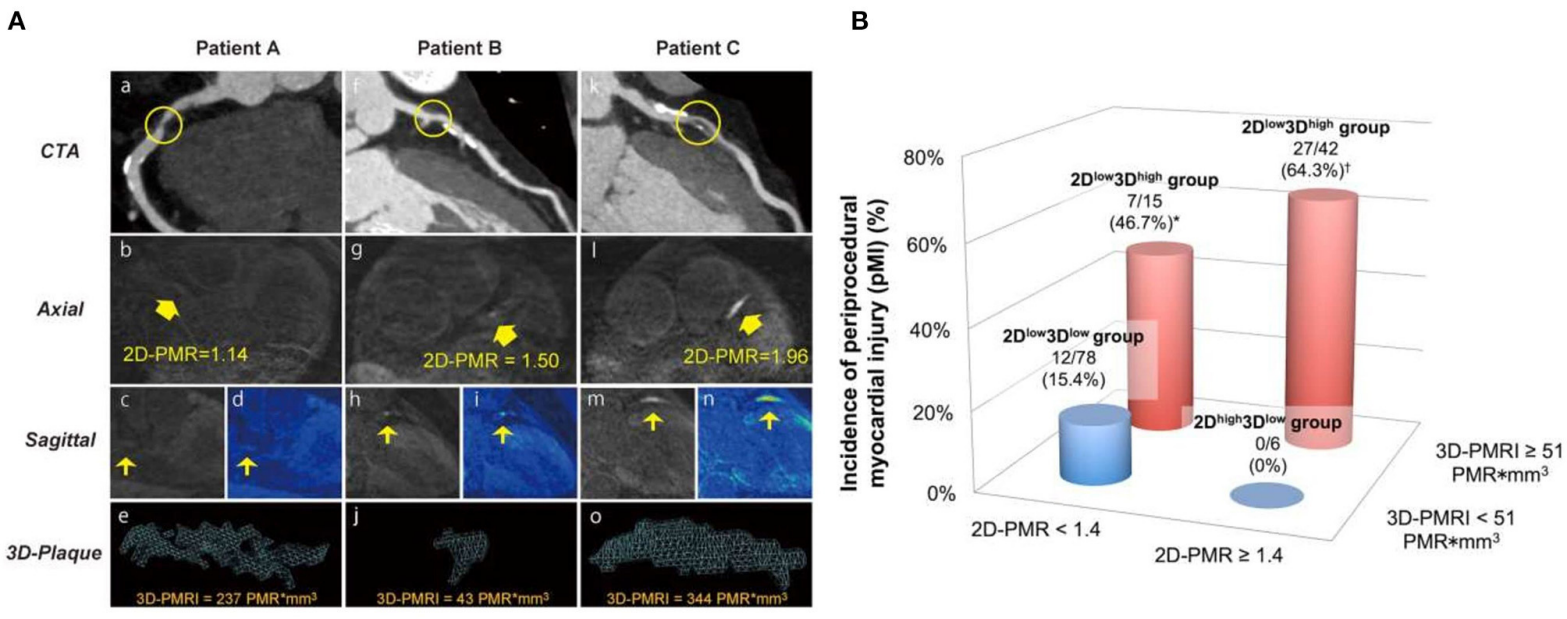
**(A)** Representative 2-dimensional and 3-dimensional plaque assessment on T1-weighted imaging. Coronary plaques with 2D^low^3D^high^ in the proximal right coronary artery (2D-PMR, 1.14; 3Di-PMR, 237 PMR*mm^3^; Patient A: a–e), 2D^high^3D^low^ in the proximal left anterior descending artery (LAD) (2D-PMR, 1.50; 3Di-PMR, 43 PMR*mm^3^; Patient B: f–j), and 2D^high^3D^high^ in the proximal LAD (2D-PMR, 1.96; 3Di-PMR, 344 PMR*mm^3^; Patient C: k–o). Computed tomography angiography (CTA) images (a, f, k), and axial images (b, g, l), sagittal images (c, h, m), colour maps (d, I, n), and 3D region of interests (3D plaque: e, j, n) on T1w images are shown. Yellow circles indicate percutaneous coronary intervention target lesion sites on CTA. Yellow arrows indicate lesions on T1w imaging corresponding to a lesion on angiography that underwent intervention. **(B)** Incidence of periprocedural myocardial injury (pMI) based on 3Di-PMR and 2D-PMR cutoff values. The red and blue bars represent patients with 3Di-PMR ≥ 51 PMR*mm^3^ and <51 PMR*mm^3^, respectively. *P* < 0.001 based on the chi-squared test. *P = 0.006 vs. 2D^high^3D^low^ group. *P* < 0.001 vs. 2D^low^3D^low^ group, and *P* = 0.003 vs. 2D^high^3D^low^group. Adapted with permission from Hosoda et al. (90).

Our understanding of CAD and CCS is rapidly evolving. The COURAGE trial highlighted the limitations of coronary luminography as a standalone prognostic measure of CAD, with no significant difference in revascularisation vs. best medical therapy on hard end point outcomes such as death or myocardial infarction in patients that were angiographically identified to have significant CAD (83). The FAME trials brought into sharp focus the prognostic benefits of functional physiological assessment of CAD, demonstrating supplementary benefits in terms of death, myocardial infarction and urgent revascularisation when coronary intervention was based on functional assessment with FFR compared with invasive angiography alone or medical therapy alone (7, 84). However, in the recently published ISCHEMIA trial of 5,179 patients with CCS, there was no prognostic or indeed symptomatic benefit of functionally guided revascularisation vs. medical therapy alone after 4 years of follow up, although patients with left main stenosis were excluded from this study (85). Therefore, academic focus is on finding a more specific marker in patients with CCS who are more likely to have a prognostically worse clinical outcome (86–88). Identification of high-risk coronary plaques by CMR has been shown to be an independent and prognostically significant marker of CAD in patients with CCS, in addition to or indeed regardless of coronary luminal stenosis (89). It has also recently been shown to predict periprocedural myocardial injury (Figure 12) (90). CMR has the capability to detect vulnerable coronary plaques by taking advantage of the intrinsic T1 shortening of plaque components (e.g., intraplaque haemorrhage, thrombus, and lipid core), both with and without the need for contrast agents (91–95).

**Figure 13:**
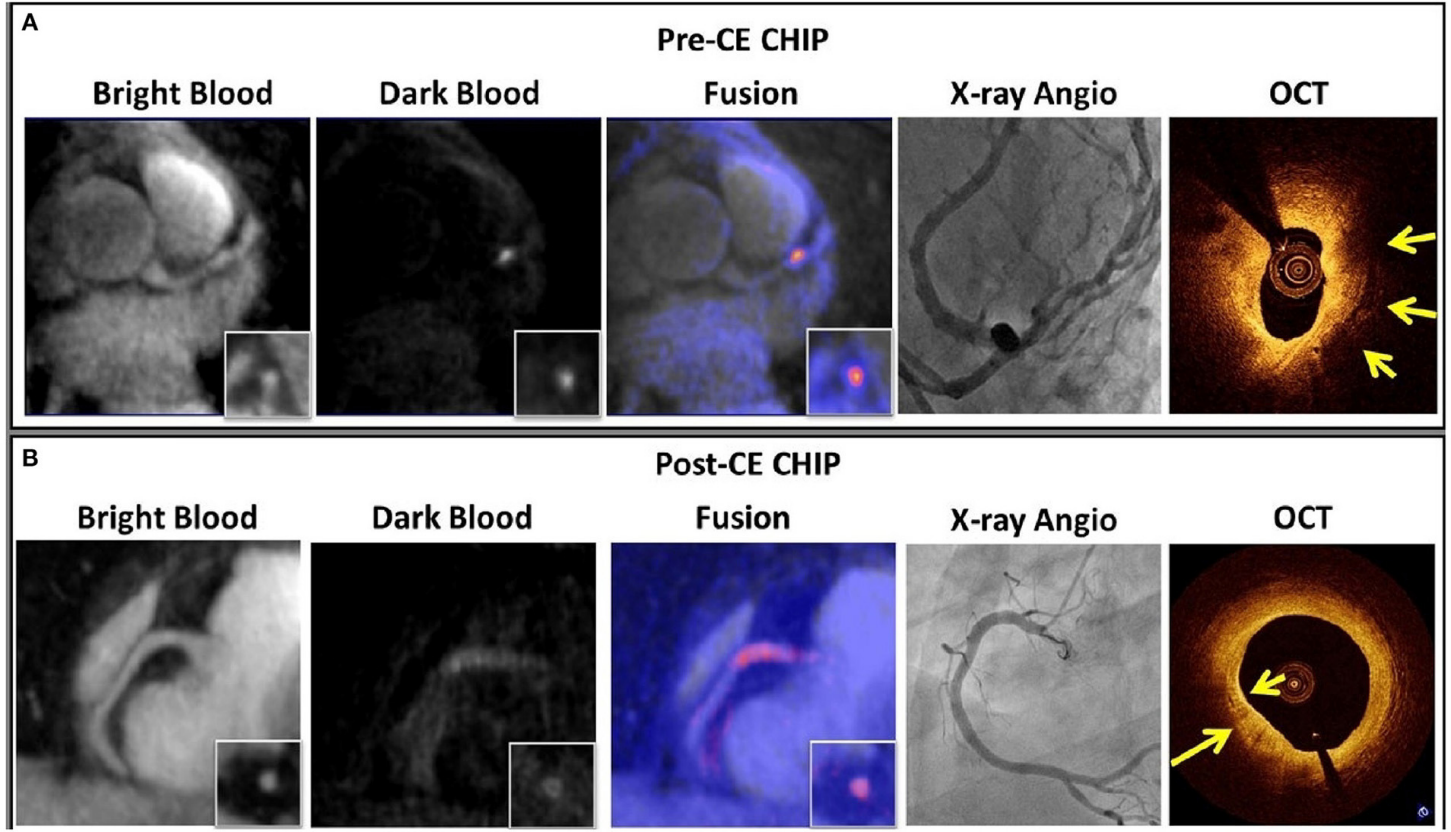
**(A)** An example of pre-CE CHIP was found in the middle LAD visualised on the dark blood images and fused with the bright-blood scan. XA showed significant stenosis (70%) at that location. OCT showed large signal-poor area suggestive of possible lipid core and/or intra-plaque haemorrhage (yellow arrow). **(B)** An example of post-CE CHIP with diffuse wall enhancement at proximal RCA as localized on the bright-blood images. XA showed only mild stenosis (30%) at that location. OCT showed strong multi-focal back reflections and signal heterogeneity within the overlaying tissue suggestive of high macrophage density (yellow arrows). CE, contrast enhancement; CHIP, coronary hyper-intensive plaques; XA, X-ray angiography; OCT, optical coherence tomography. Adapted with permission from Xie et al. (96).

**Figure 14:**
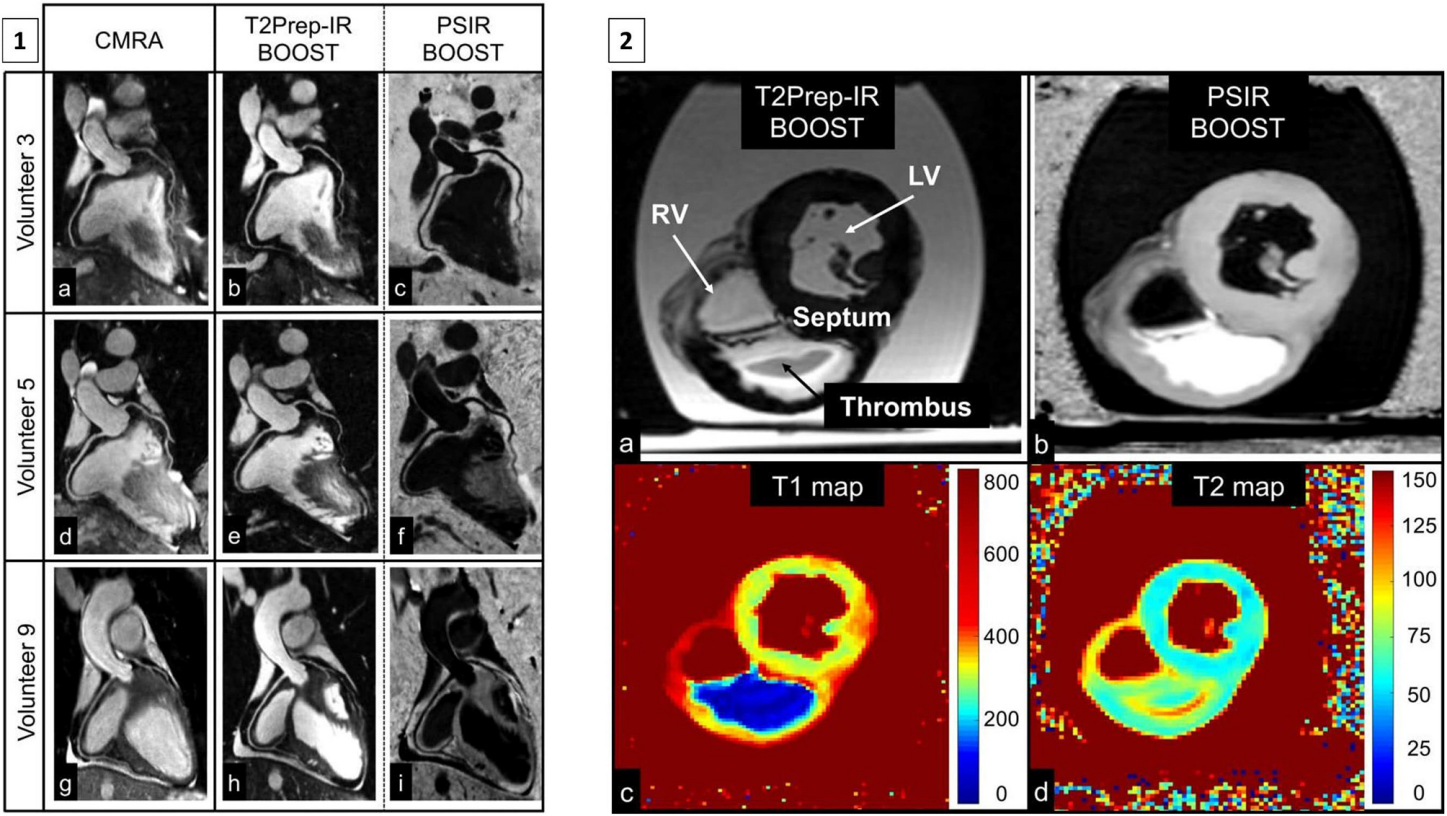
(**1**) Reformatted coronary depiction in three representative healthy volunteers obtained with a conventional T_2_-prepared bright-blood CMRA acquisition (a, d, g) and the proposed BOOST sequence for simultaneous bright-blood (T_2_Prep-IR BOOST datasets in b, e, h) and black-blood (PSIR BOOST datasets in c, f, i) whole-heart MRI. Quantified CNR_blood−myo_ significantly improved with the proposed T_2_Prep-IR BOOST approach in comparison to the conventional CMRA, thus leading to a higher quantified coronary percentage vessel sharpness (%VS) for both right and left coronary arteries. In the PSIR BOOST images in (c, f, i), the efficacy of blood signal suppression can be appreciated along multiple portions of the coronary tree. (**2)** MRI images obtained in the *ex vivo* pig heart. All the images depict a short-axis view at the midventricular level. Images acquired with the proposed BOOST sequence are reported in (a) (bright-blood T_2_Prep-IR dataset) and in (b) (black-blood PSIR-like reconstruction). RV, LV, thrombus, and interventricular septum are indicated. The black-blood reconstruction (b) clearly enhances the signal from the thrombus when compared to the bright-blood dataset (a). Furthermore, 2D T_1_ (c) and T_2_ (d) mapping techniques were acquired. The *ex vivo* thrombus is characterized by a relatively short T_1_ and T_2_. BOOST, Bright-blood and black-blOOd phase SensiTive; IR, inversion recovery; myo, myocardium; PSIR, phase-sensitive inversion recovery; T_2_Prep, T_2_ prepared; LV, left ventricular cavity; RV, right ventricular cavity. Adapted with permission from Ginami et al. (98).

Despite this, MR coronary plaque imaging is constrained by comparable technical challenges to CMRA, e.g., respiratory motion, prohibitively long and unpredictable acquisition time, plaque to coronary artery misalignment due to the sequential nature of data acquisition. Recently, a novel framework (CATCH) has been proposed to overcome some of these limitations for MR coronary plaque imaging. This framework assembles advanced motion correction techniques outlined above to facilitate simultaneous multi contrast bright and black blood coronary artery angiography and visualisation of vulnerable coronary plaque (Figure 13) (96, 97). However, there is incomplete overlap of respiratory motion parameters between the bright-blood and black-blood datasets, potentially resulting in residual coronary plaque-misregistration. More recently, another framework has been proposed (BOOST) for simultaneous coronary angiography and thrombus/intraplaque haemorrhage visualisation (Figure 14) (98). This framework works by acquiring two differently weighted bright-blood datasets in alternate heart beats, which are subsequently processed in a phase-sensitive inversion recovery (PSIR)-like reconstruction to produce a third, black-blood dataset. The two bright-blood datasets enable respiratory motion to be independently estimated and corrected, which reduces the likelihood of misregistration artefacts. It also uses the iNAV technology with a highly undersampled golden angle Cartesian acquisition, which enables 100% respiratory scan efficiency, 2D rigid translational motion correction as well as 3D non-rigid motion estimation. Traditional reconstruction frameworks (e.g., iterative sense, compressed sensing or low rank patched based frameworks) or deep learning neural network reconstruction can be applied. These frameworks require further extensive clinical validation, ideally against intravascular imaging modalities such as intravascular ultrasound or optical coherence tomography.

It is also possible to directly target specific components of the atherosclerotic plaque (99–101). Various MR specific contrast agents are currently in development which can target molecular components that are involved in atherosclerotic plaque initiation, progression, instability and plaque rupture, such as elastin (102), tropoelastin, collagen (103, 104), fibrin (105), and matrix metalloproteinases (106). Whilst these frameworks have shown great promise in pre-clinical feasibility studies, the challenge is to safely replicate these findings in clinical validation studies.

## Conclusions

Chronic coronary syndrome is a progressive and multifaceted condition associated with coronary artery atherosclerosis, which manifests clinically as either angina, heart failure and gradual left ventricular dysfunction, arrhythmia and/or acute myocardial infarction. The timely recognition of CCS enables bespoke risk assessment of patients, prompt initiation of therapeutical intervention and long-term monitoring of potential complications. Amongst a maelstrom of diagnostic imaging modalities for CAD, CMRA could potentially emerge as a viable, versatile, reproducible and non-invasive imaging modality for the assessment of CCS, which is free of ionising radiation, iodinated contrast agents and can be performed without gadolinium contrast enhancement. These characteristics would be ideally suited for repeat imaging of patients to monitor disease progression and gauge response to treatment. Furthermore, it can be combined with myocardial volumetric assessment, tissue characterisation, perfusion and coronary plaque assessment to enable comprehensive assessment of ischaemic heart disease.

## Author Contributions

RH drafting and editing of the manuscript. CM, GC, RK, WK, CP, and RB reviewing and editing of manuscript. All authors contributed to the article and approved the submitted version.

## Conflict of Interest

The authors declare that the research was conducted in the absence of any commercial or financial relationships that could be construed as a potential conflict of interest.

## Publisher's Note

All claims expressed in this article are solely those of the authors and do not necessarily represent those of their affiliated organizations, or those of the publisher, the editors and the reviewers. Any product that may be evaluated in this article, or claim that may be made by its manufacturer, is not guaranteed or endorsed by the publisher.
